# MG132 Induces Progerin Clearance and Improves Disease Phenotypes in HGPS-like Patients’ Cells

**DOI:** 10.3390/cells11040610

**Published:** 2022-02-10

**Authors:** Karim Harhouri, Pierre Cau, Frank Casey, Koffi Mawuse Guedenon, Yassamine Doubaj, Lionel Van Maldergem, Gerardo Mejia-Baltodano, Catherine Bartoli, Annachiara De Sandre-Giovannoli, Nicolas Lévy

**Affiliations:** 1 Marseille Medical Genetics (MMG), INSERM U 1251, Aix Marseille Université, 13005 Marseille, France; Karim.HARHOURI@univ-amu.fr (K.H.); catherine.bartoli@univ-amu.fr (C.B.); annachiara.desandre-giovannoli@univ-amu.fr (A.D.S.-G.); 2 Progelife, 13002 Marseille, France; pierre.cau@progelife.com; 3 Royal Belfast, Pediatric Cardiology, Hospital for Sick Children, Belfast BT9 7AB, Northern Ireland, UK; frank.casey@belfasttrust.hscni.net; 4 CHU Sylvanus Olympio de Lomé, Unité de Génétique Humaine, Lomé BP 1515, Togo; julesblack@yahoo.fr; 5 Département de Génétique Médicale, Institut National d’Hygiène, Rabat 11400, Morocco; y.doubaj@gmail.com; 6 Centre de Génétique Humaine, CHU Université de Franche-Comté, 25000 Besancon, France; lvanmaldergem@chu-besancon.fr; 7 Departamento de Genética, Ministerio de Salud de Nicaragua, Hospital Infantil “Manuel de Jesús Rivera”, Managua 12079, Nicaragua; mejiabaltodano@gmail.com; 8 Département de Génétique Médicale, Hôpital d’Enfants de la Timone, AP-HM, 13005 Marseille, France; 9 Biological Resource Center (CRB-TAC), Assistance Publique Hôpitaux de Marseille, La Timone Children’s Hospital, 13005 Marseille, France

**Keywords:** progeria-like, MAD-B, progerin, prelamin A Δ90, prelamin A Δ35, MG132, autophagy, inflammation

## Abstract

Progeroid syndromes (PS), including Hutchinson-Gilford Progeria Syndrome (HGPS), are premature and accelerated aging diseases, characterized by clinical features mimicking physiological aging. Most classical HGPS patients carry a de novo point mutation within exon 11 of the *LMNA* gene encoding A-type lamins. This mutation activates a cryptic splice site, leading to the production of a truncated prelamin A, called prelamin A ∆50 or progerin, that accumulates in HGPS cell nuclei and is a hallmark of the disease. Some patients with PS carry other *LMNA* mutations and are named “HGPS-like” patients. They produce progerin and/or other truncated prelamin A isoforms (∆35 and ∆90). We previously found that MG132, a proteasome inhibitor, induced progerin clearance in classical HGPS through autophagy activation and splicing regulation. Here, we show that MG132 induces aberrant prelamin A clearance and improves cellular phenotypes in HGPS-like patients’ cells other than those previously described in classical HGPS. These results provide preclinical proof of principle for the use of a promising class of molecules toward a potential therapy for children with HGPS-like or classical HGPS.

## 1. Introduction

Progeroid syndromes (PS) are a group of very rare genetic disorders associated with clinical features that mimic physiological aging. Hutchinson-Gilford Progeria Syndrome (HGPS, OMIM #176670) is the most prevalent and widely studied syndrome among PS. Estimates indicate that the prevalence of HGPS is approximately one in 4 million children [1]. HGPS is characterized by premature and accelerated aging with rapid growth retardation, thin skin, loss of subcutaneous fat, alopecia, osteoporosis, and cardiovascular disease. HGPS patients’ death occurs at the mean age of 14.6 years [2], almost exclusively due to heart attack or stroke caused by atherosclerosis. In 2003, we and others independently identified a recurrent de novo point mutation (c.1824C>T, p.G608G) in the *LMNA* gene (1q21) encoding A-type lamins as the most frequent cause of classical progeria [3,4]. In physiological conditions, *LMNA* encodes lamins A and C through alternative pre-mRNA splicing. Lamins A/C are major components of the nuclear lamina, a protein meshwork located underneath the inner membrane of the nuclear envelope and dispersed through the nuclear matrix [5]. The HGPS mutation activates a cryptic splice site in prelamin A-encoding mRNAs, mainly regulated by the serine–arginine rich splicing factor 1 (SRSF-1) [6], leading to the production of a truncated and permanently farnesylated prelamin A precursor (called progerin). Progerin cannot be properly post-translationally processed to mature lamin A and thus accumulates at the cell nuclear periphery. Progerin exerts a series of toxic, dose-dependent, dominant negative effects, including altered heterochromatin dynamics, DNA damage repair defects, chronic inflammation, proliferation slowdown, and accelerated senescence [7]. Progerin intranuclear accumulation has thus been identified as a major HGPS pathophysiological target and is being involved in most, if not all, of the nine hallmarks of physiological aging [8].

A wide spectrum of treatment strategies with different specificities, targeting several processes, has been proposed to correct the defects in HGPS: (i) to “repair” the disease-causing mutation; (ii) to block pre-mRNA aberrant splicing leading to progerin mRNA production; (iii) to reduce the toxicity of isoprenylated and methylated progerin; (iv) to induce progerin clearance; (v) to decrease the noxious downstream effects linked to progerin accumulation [9,10]. However, targeting only one pathophysiological event of progeria and related diseases would not result in a reversal of the pathological phenotypes of such segmental disorders that affect multiple tissues; hence, therapeutic approaches targeting several mechanisms triggering the disease needed to be envisaged for all related syndromes having common pathophysiological mechanisms. These approaches should succeed in lowering the amount of aberrant prelamin A isoforms at different levels, including their decreased production, increased degradation, as well as counteracting downstream toxic effects. In a previous study, we demonstrated that proteasome inhibitor MG132, not yet FDA/EMA-approved, induces significant progerin inhibition through a dual action: MG132 reduces progerin production through the downregulation of SRSF-1 and SRSF-5, controlling prelamin A mRNA aberrant splicing, and induces progerin degradation through macroautophagy. Macroautophagy activation in HGPS cells is supported by the increased ratios of LC3B-II/LC3B-I, progerin delocalization into cytoplasmic autophagic vacuoles containing the autophagic markers p62, LAMP-2, and LC3B, increased autophagic transcript levels using RNA-seq experiments, and partial restoration of progerin levels in the presence of autophagy inhibitors: chloroquine or bafilomycin A1. Otherwise, it is known that impairment of the ubiquitin-proteasome system is compensated by the activation of autophagy [11,12,13]. MG132 treatment improves cellular HGPS phenotypes in vitro, and injection of the drug in the skeletal muscle of a mouse model of progeria (*Lmna^G609G/G609G^*) locally reduced SRSF-1 expression and progerin levels [14].

Besides typical HGPS, there are other forms of progeroid syndromes characterized by signs of aging, called HGPS-like. Most of the HGPS-like patients carry mutations near the donor splice site of exon 11, causing the production of variable quantities of aberrant prelamin A isoforms. In particular, the prelamin A ∆90 transcript excludes the 270 nucleotides of exon 11 because of the abolition of the normal donor splice site. The resulting deletion is predicted to preserve the prelamin A open reading frame (r.[=, 1699_1968del], p.(Gly567_Gln656del)). The mutation responsible for the production of prelamin A ∆35 generates a conservative substitution of serine with threonine and activates a cryptic splice site, resulting in the expression of a truncated prelamin A lacking 35 amino acids (r.[=, 1864_1968del], p.[Thr623Ser, Val622_Gln656del]) [15,16]. The group of progeroid syndromes also includes patients affected with Mandibuloacral Dysplasia type B (MAD-B), carrying a homozygous mutation in ZMPSTE24, encoding the FACE1 protease involved in prelamin A maturation and leading to the accumulation of wild-type farnesylated Prelamin A. We hypothesized that MG132 could also be beneficial for HGPS-like patients whose cells express WT prelamin A, prelamin A ∆35, prelamin A ∆50, or/and prelamin A ∆90. Here, we test the effects of MG132 on HGPS-like and MAD-B cells and characterize the drug effect on aberrant prelamin A isoform clearance as well as the improvement of cell phenotypes.

## 2. Materials and Methods

### 2.1. Patients and Samples

Samples were collected from eight patients affected with typical HGPS, HGPS-like, or MAD-B syndromes, showing different genomic pathogenic variants, variable clinical phenotypes and disease severity but all having in common the accumulation of aberrant and toxic prelamin A isoforms. Patients were from the USA (HGPS-L1), the UK (HGPS-L2 and HGPS-L6), Greece (HGPS-L3), Nicaragua (HGPS-L4), France (HGPS-L5), and Togo (MAD-B). Informed consent was obtained from the patients or the parents of minor patients included in this work, allowing studies on their cells as part of a diagnosis and research program, complying with the ethical guidelines of the institutions involved. Parents also gave written consent for picture publication, including uncovered faces. The dermal fibroblast cell line from patient HGPS-L 1 was provided by the Progeria Research Foundation Cell and Tissue Bank under the cell line name PSADFN386 (https://www.progeriaresearch.org/wp-content/uploads/2021/09/PRF-AVAILABLE-CELL-LINES-09-02-21.pdf, accessed on 6 January 2022); the other human dermal fibroblast cell lines were issued from a skin biopsy, prepared and stored by the certified Biological Resource Center (CRB AP-HM Biobank; NF S96-900 & ISO 9001 v2015 Certifications), Department of Medical Genetics, La Timone Hospital of Marseille, according to French regulations. The fibroblast cell lines used belong to a biological sample collection declared to the French Ministry of Health (declaration number DC-2008-429), whose use for research purposes was authorized by the French Ministry of Education, Research, and Innovation (authorization number AC-2011-1312; AC-2017-2986).

### 2.2. Genomic Characterization of LMNA Variants

All the patients included in this work, except for patient HGPS-L4, have already been described [15,16]. Patient HGPS-L4 is first described in this work, and her genomic characterization was performed as described in [15] upon Sanger sequencing of the *LMNA* gene, which was directly performed in a diagnosis setting upon clinical suspicion. Briefly, Primer-3 designed specific primers were used for the PCR amplification of each *LMNA* coding exon. PCR products were examined by agarose gel electrophoresis and then subjected to Sanger sequencing. Sequencher 4.8 (Gene Codes Corp., Ann Arbor, MI, USA) was used for the interpretation of sequence variants. Sequence variants are described following the Human Genome Variations Society Guidelines, available at https://varnomen.hgvs.org/, accessed on 6 January 2022. *LMNA* and *ZMPSTE24/FACE*1 variants are respectively described relative to transcript reference sequences NM_170707.3 and NM_005857.5.

### 2.3. Cell Culture

Human dermal fibroblasts (established from a skin biopsy) were cultured in Dulbecco’s modified Eagle’s medium (Thermo Fisher Scientific, Waltham, MA, USA) supplemented with 15% fetal bovine serum (Thermo Fisher Scientific), 2 mM L-glutamine (Thermo Fisher Scientific), and penicillin–streptomycin (Thermo Fisher Scientific) at 37 °C in a humidified atmosphere containing 5% CO_2_. Testing for mycoplasma contamination was performed monthly. Fibroblasts were treated with media containing 500 nM or 5 µM MG132 (474790, Merck, Fontenay sous Bois, Île-de-France, France), 10 ng/mL TNFα (210-TA, R&D Systems, Minneapolis, MN, USA), a combination of 500 nM MG132 and 10 ng/mL TNFα or with media containing the same volume of DMSO (vehicle control). The experiments were performed on fibroblasts of patients and healthy subjects matched for age and passage number.

### 2.4. RNA Sequencing (ArrayExpress Accession Number: E-MTAB-5807)

RNA sequencing was performed by IntegraGen (5, Rue de Henri Desbruères 91000 Evry, Région d’ Île-de-France, France). RNA samples were used to generate sequencing libraries with the TruSeq Stranded mRNA Sample Prep’ Illumina^®^. The libraries were sequenced on an Illumina HiSeq 4000 sequencer, yielding approximately 35 million 2 × 75-bp paired-end reads.

#### 2.4.1. Quality Control

Quality of reads was assessed for each sample using FastQC (http://www.bioinformatics.babraham.ac.uk/projects/fastqc/, accessed on 16 December 2021).Sequence alignment and quantification of gene expression: A subset of 500,000 reads from each Fastq file was aligned to the reference human genome hg38 with TopHat2 to determine insert sizes with Picard. Full Fastq files were aligned to the reference human genome hg38 with TopHat2 (-p 24 -r 150 -g 2 -library-type fr-firststrand). Reads mapping to multiple locations were removed. Gene expression was quantified using two non-overlapping transcriptome annotations: the full Gencode v25 annotation as well as a complementary lncRNA annotation. HTSeq was used to obtain the number of reads associated with each gene in the Gencode v25 database (restricted to protein-coding genes, antisense, and lincRNAs) and each gene in the additional lncRNA database. The Bioconductor DESeq package was used to import raw HTSeq counts for each sample into R statistical software and extract the count matrix. After normalizing for library size, the count matrix was normalized by the coding length of genes to compute FPKM scores (number of fragments per kilobase of exon model and millions of mapped reads). Bigwig visualization files were generated using the bam2wig Python script.

#### 2.4.2. Unsupervised Analysis

The Bioconductor DESeq package was used to import raw HTSeq counts into R statistical software, to obtain size factors, and to calculate a variance stabilizing transformation (VST) from the fitted dispersion–mean relations to normalize the count data. The normalized expression matrix from the 1000 most variant genes (based on standard deviation) was used to classify the samples according to their gene expression patterns using principal component analysis (PCA) and hierarchical clustering.

#### 2.4.3. Differential Expression Analysis

The Bioconductor DESeq package was used to import raw HTSeq counts into R statistical software, obtain size factors and dispersion estimates, and test differential expression. Only genes expressed in at least one sample (FPKM ≥ 0.1) were tested to improve the statistical power of the analysis. A q-value threshold of ≤0.05 was applied to define differentially expressed genes.

### 2.5. RNA Isolation, Reverse Transcription, and Real-Time PCR

Total RNA was isolated using the RNeasy plus extraction kit (Qiagen, Valencia, CA, USA), and the samples were quantified and evaluated for purity (260 nm/280 nm ratio) with a NanoDrop ND-1000 spectrophotometer (Thermo Fisher Scientific). Then, 1 µg of RNA was reverse transcribed using a SuperScript IV Reverse Transcriptase Kit (Thermo Fisher Scientific, Waltham, MA, USA). Real-time PCR amplification was carried out with the TaqMan Gene Expression Master Mix (Thermo Fisher Scientific, Waltham, MA, USA) on a LightCycler 480 (Roche, Germany) using predesigned primers for RPS13 (hs-01011487_g1), progerin (F: ACTGCAGCAGCTCGGGG. R: TCTGGGGGCTCTGGGC and probe: CGCTGAGTACAACCT), lamin A (F: TCTTCTGCCTCCAGTGTCACG. R: AGTTCTGGGGGCTCTGGGT and probe: ACTCGCAGCTACCG), and lamin C (F: CAACTCCACTGGGGAAGAAGTG. R: CGGCGGCTACCACTCAC and probe: ATGCGCAAGCTGGTG), Prelamin A ∆90 (F: CGAGGATGAGGATGGAGATGA. R: CAGGTCCCAGATTACATGATGCT, overlapping exons 10 and 12 and probe: CACCACAGCCCCCAGA), and Prelamin A ∆35 (F: ACTGCAGCAGCTCGGGG. R: AGTTCTGGGGGCTCGTGAC Probe: CGCTGAGTACAACCT) (Applied Biosystems, Foster, CA, USA). The gene expression of IL-1α, IL-1β, IL-6, IL-8, TNFα, IFN-β, EGFR, NFκB1, NFκB2, NFκBIα, Rel A, Cox-2, and the 18S rRNA control was assessed through real-time PCR using TaqMan^®^ Gene Expression Array Plates (ThermoFisher Scientific) containing predesigned, gene-specific primers and probes (Table 1). All qPCRs were performed using the program: UNG incubation at 50 °C for 2 min, initial denaturation at 95 °C for 10 min, 40 cycles of amplification:denaturation at 95 °C for 15 s, and annealing at 60 °C for 1 min. All PCRs were performed in triplicate. Threshold cycle (Ct) values were used to calculate relative mRNA expression by the 2-∆∆Ct relative quantification method with normalization to RPS13 expression.

### 2.6. Western Blot

Total fibroblast proteins were extracted in 200 µL of NP40 Cell Lysis Buffer (Thermo Fisher Scientific, Waltham, MA, USA) containing Protease and Phosphatase Inhibitor Cocktail (Thermo Fisher Scientific, Waltham, MA, USA). Cells were sonicated twice (30 s each), incubated at 4 °C for 30 min, and then centrifuged at 10,000× *g* for 10 min. Protein concentration was evaluated with the bicinchoninic acid technique (Pierce BCA Protein Assay Kit, Thermo Fisher Scientific); absorbance at 562 nm was measured using Nanodrop 1000 (Thermo Fisher Scientific). Equal amounts of proteins (40 µg) were loaded onto 10% Tris-glycine gel (CriterionTM XT precast gel) using the XT Tricine Running Buffer (Bio-Rad, Hercules, CA 94547, USA). After electrophoresis, gels were electrotransferred onto Immobilon-FL polyvinylidene fluoride membranes (Merck, Fontenay sous Bois, Île-de-France, France), blocked in Odyssey blocking buffer (Eurobio Scientific, les Ulis, France) diluted 1:1 in PBS for 1 h at room temperature, and incubated overnight at 4 °C or 2 h at room temperature with various primary antibodies. Blots were washed with TBS-T buffer (20 mM tris (pH 7.4), 150 mM NaCl, and 0.05% Tween 20) and incubated with 1:10,000 IR-Dye 800-conjugated secondary donkey anti-goat or IR-Dye 700-conjugated secondary anti-mouse antibodies (LI-COR Biosciences, Lincoln, NE, USA) in Odyssey blocking buffer. For IR-Dye 800 and IR-Dye 700 detection, an odyssey Infrared Imaging System (LI-COR Biosciences) was used. GAPDH or actin was used as a total cellular protein loading control.

### 2.7. Fluorescence Microscopy

Fibroblasts were seeded into 4-well cell culture slides (Lab-tek, SPL Life Sciences, Pocheon-si, Gyeonggi-do, Korea), fixed with 4% paraformaldehyde, washed with PBS, and permeabilized with 0.5% Triton X-100 for 15 min. After PBS washing, slides were incubated with 1% bovine serum albumin for 30 min at room temperature before adding the primary antibodies for 3 h at 37 °C or overnight at 4 °C. After washing, the cells were then incubated with secondary antibodies (A11001, A11058, Thermo Fisher Scientific; 1/400) for 1 h at room temperature. Nuclei were stained with DAPI (50 ng/mL) and diluted in Vectashield (Abcys, Paris, France) for 10 min at RT. The stained cells were observed with a Zeiss LSM 800 Confocal Microscope using Zen 2.3 software (Rueil Malmaison, France). All antibodies were tested in individual staining reactions for their specificity. Controls without a primary antibody were all negative.

### 2.8. Antibodies

Antibodies used in the study included: a rabbit anti-lamin A/C polyclonal antibody that reacts with lamin A, lamin C, and progerin (#SC-20681, used at 1:1000 dilution for the Western blot analyses, Santa Cruz Biotechnology Inc. Dallas, TX, USA); a goat anti-prelamin A polyclonal antibody (#sc-6214 used at 1:1000 dilution for the Western blot analyses, Santa Cruz Biotechnology Inc.); a mouse anti-actin monoclonal antibody (#MAB1501R, used at 1:5000 dilution for the Western blot analyses, Merck, Fontenay sous Bois, Île-de-France, France); a mouse anti-glyceraldehyde-3-phosphate dehydrogenase monoclonal antibody (#MAB374, used at 1:10,000 for the Western blot analyses, Merck); a rabbit anti-LC3B polyclonal antibody (#2775, used at 1:1000 for the Western blot analyses, Cell Signaling Technology/Ozyme, Saint-Cyr-L’École, France); a rabbit anti-IκBα monoclonal antibody (#4812, used at 1:1000 dilution for the Western blot analyses, Cell Signaling/Ozyme); a rabbit anti-histone H3 (Tri-Me-K9) polyclonal antibody (#ab8898, used at 1:100 for immunofluorescence labeling, Abcam, Paris, France); a rabbit anti-lamin-B1 polyclonal antibody (#ab 16048, used at 1:100 for immunofluorescence labeling, Abcam); a rabbit anti-LAP2a polyclonal antibody (#ab5162, used at 1:100 for immunofluorescence labeling, Abcam); a mouse anti-γH2A.X (phospho S139) monoclonal antibody (#ab26350, used at 1:200 for immunofluorescence labeling, Abcam); a rabbit anti-53BP1 polyclonal antibody (#NB100-304, used at 1:1000 for immunofluorescence labeling, Novus Biologicals, Cambridge, UK).

### 2.9. Measurement of Senescence

Senescence was measured using 2 assays: 1/Beta-Glo Assay Kit (# E4720, Promega, Charbonnières-les-Bains, France), according to the manufacturer’s instructions and utilizing a luciferin-galactoside substrate (6-O-β galactopyranosylluciferin). This substrate is cleaved by β-galactosidase to form luciferin and galactose. The luciferin is then utilized in a firefly luciferase reaction to generate a bright luminescent signal, determined as RLUs using a GloMax-Multi Detection System: Luminometer (Promega). 2/Colorimetric detection of senescence-associated β galactosidase, following the manufacturer’s protocol (#9860, Cell Signaling/Ozyme, Saint-Cyr-L’École, France). Cells were seeded in 4 chamber-wells slides (SPL Lifesciences, Pocheon-si, Gyeonggi-do, Korea), washed with PBS, and fixed in Fixative solution (1/10 dilution) for 15 min at RT. Cells were washed in PBS and stained overnight at 37 °C with β-galactosidase staining solution. Stained samples were visualized using a bright-field microscope (Leica, Wetzlar, Germany).

### 2.10. Proliferation Assay

Cell proliferation rate was measured with a BrdU Cell Proliferation ELISA Kit (Abcam, Paris, France), according to the manufacturer’s instructions. Absorbance was monitored with a GloMax-Multi Detection System: Luminometer (Promega, Charbonnières-les-Bains, France).

### 2.11. Wound Healing Assay

A reproducible wound was performed with a pipette tip on a confluent monolayer of WT, HGPS, HGPS-like, and MAD-B fibroblasts cultured on 96-well plates. The medium was removed, and cells were incubated for 6 h with medium containing 500 nM MG132 or equal volume DMSO. The surface of the wound was acquired with a Zeiss Axio Observer using Zen 2.3 pro-software (Rueil Malmaison, France) and measured with ImageJ software v1.52K (NIH, Bethesda, MD, USA). Results were expressed as a percentage of the area of the original wound and normalized to DMSO-treated cells, considered as 100%.

### 2.12. Multi-Analyte ELISA Array

Multi-array ELISA was performed using the Multi-Analyte ELISArray Kit (Qiagen, Hilden, Germany) according to the manufacturer’s instructions. In brief, the supernatants were centrifuged for 10 min at 1000× *g* to remove any particulate material. Then, 50 µL of each experimental sample was added to the array coated with specific cytokine capture antibodies: IL-1α IL-1β, IL-2, IL-4, IL-6, IL-8, IL-10, IL-17α, TNF-α, IFN-γ, TGFβ, and GROa and incubated at room temperature (RT) for 2 h. After three washes, 100 μL of the diluted biotinylated detection antibodies were added to the appropriate wells of the ELISA plate and incubated in the dark for 1 h at RT. The plate was washed three times, and 100 μL of diluted Avidin-horseradish peroxidase (HRP) were added into all wells and incubated in the dark for 30 min at RT. Development and stop solutions were added, followed by detection of absorbance at 450 on a Luminometer: GloMax-Multi Detection System (Promega, Charbonnières-les-Bains, France).

### 2.13. Statistics

Statistical analyses were performed with the GraphPad Prism software. Differences between groups were assayed using a two-tailed Student’s *t*-test. In all cases, the experimental data were assumed to fulfill *t*-test requirements (normal distribution and similar variance); in those cases, where the assumption of the *t*-test was not valid, a nonparametric statistical method was used (Mann–Whitney test). A *p*-value less than 0.05 was considered significant. Error bars indicate the standard error of the mean.

## 3. Results

### 3.1. Patients’ Molecular and Clinical Features

**Figure 1:**
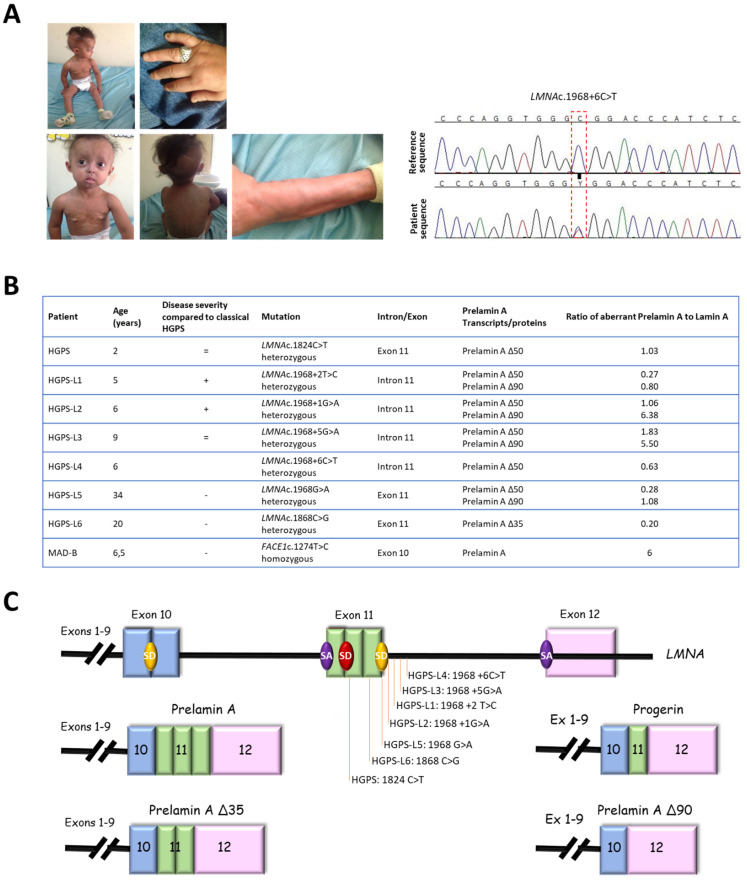
Clinical and molecular description of cell lines. (**A**) Pictures and electropherograms of patient HGPS-L4 at age 6 years showing progeroid features, including sparse hair and eyebrows, small chin, a thin nose, prominent nipples, dyspigmentation with hyper/hypo-pigmented areas, and sclerodermatous changes on her chest. The heterozygous c.1968+6C>T *LMNA* mutation was confirmed by Sanger sequencing. (**B**) Characterization of *LMNA* and FACE1 (ZMPSTE24) gene mutations in HGPS-like and MAD-B patients eliciting aberrant prelamin A splicing or wild-type prelamin A accumulation. Variable disease severities compared to classical HGPS are indicated with “+”: more, “− “: less, or “=”: equal severity. The ratios of aberrant prelamin A to lamin A isoforms are shown, issued from Western blot data, except for prelamin A, against which no antibodies are available, and so were determined based on the transcript levels. (**C**) Locations of LMNA mutations and schematic representation of the aberrant prelamin A isoforms. SD: splice donor site, SA: splice acceptor site.

Patients included in this study showed variable disease severity compared to classical HGPS but presented with similar phenotypes, including growth retardation, hair loss, prominent forehead, prominent superficial veins, thin skin, loss of subcutaneous fat, and lipodystrophy (Figure 1A). HGPS-like patients were previously reported to present distinct aberrant splicing patterns of prelamin A pre-mRNAs due to mutations located around the exon 11 donor splice site (Figure 1B,C) [16]. Briefly, patient HGPS-L1 [16] carrying the *LMNA* heterozygous c.1968+2T>C mutation, was referred to our center at the age of five years. She was diagnosed with the disease when she was 10 months old, presenting with a typical HGPS clinical phenotype, including frontal bossing, prominent veins on her scalp and forehead, sparse hair, micrognathism with delayed dentition, growth retardation (since birth, her length varied from the 2nd to the 10th centiles for age; the weight was stably < 3rd centile for age), subcutaneous lipoatrophy, dry skin with pigmentary changes on the neck and trunk, acroosteolyses with the onychodystrophy of hands and feet; laboratory findings have evidenced recurrent thrombocytosis (480–535 k/µL; normal values: 140–450 k/µL), elevated transaminases, glucose, calcium, and phosphorus, as already observed in classical HGPS patients [17]. Patient HGPS-L2 [16], carrying the heterozygous *LMNA* c.1968+1G>A mutation, showed a very similar progeroid laminopathy, though evolving more severely. She was diagnosed at nine months of age and already showed contractions of her ankles, knees, and wrist. She subsequently developed arthritis on several articulations. Her feeding was poor, and she had frequent constipation episodes. She had a bilateral hip dislocation, and, at the age of three years, she suffered from a femur fracture. At age six, she suffered from tachycardia together with sudden right arm paresis; cerebral CT scan/MRI showed multiple micro-infarcts, including recent and old ones, while echocardiography showed left ventricular thickening. After partial recovery from stroke, she suffered from a chest infection together with painful nail infections. Patients HGPS-L3 (*LMNA* heterozygous c.1968+5G>A), HGPS-L6 (*LMNA* heterozygous c.1868C>G), and HGPS-L5 (*LMNA* heterozygous c.1968G>A) were previously reported by Barthelemy et al. [15]. Patient HGPS-L4 (*LMNA* heterozygous c.1968+6C>T) is first reported in this work. She was referred to our clinics at age 4 years and presented with sparse hair and eyebrows, a small chin, a thin nose, prominent nipples, dyspigmentation with hyper/hypo-pigmented areas, and sclerodermatous changes on her chest. The MAD-B patient was first referred to us at age 6.5 years. She presented with growth retardation, exophtalmia, low-set ears, retro-micrognathism (mandibular hypoplasia), sparse hair, and thin, dry skin with hypopigmented lesions, especially on the trunk. Subcutaneous lipoatrophy gave her a muscular pseudo-hypertrophic appearance. Molecular genetic diagnosis allowed the identification of a new homozygous mutation in the ZMPSTE24/FACE1 gene’s exon 10: c.1274T>C, p.(Leu425Pro), confirming the B-type mandibuloacral dysplasia phenotype in the patient [16,18].

### 3.2. MG132 Reduces Aberrant Prelamin A Levels in HGPS-like and MAD-B Fibroblasts

**Figure 2:**
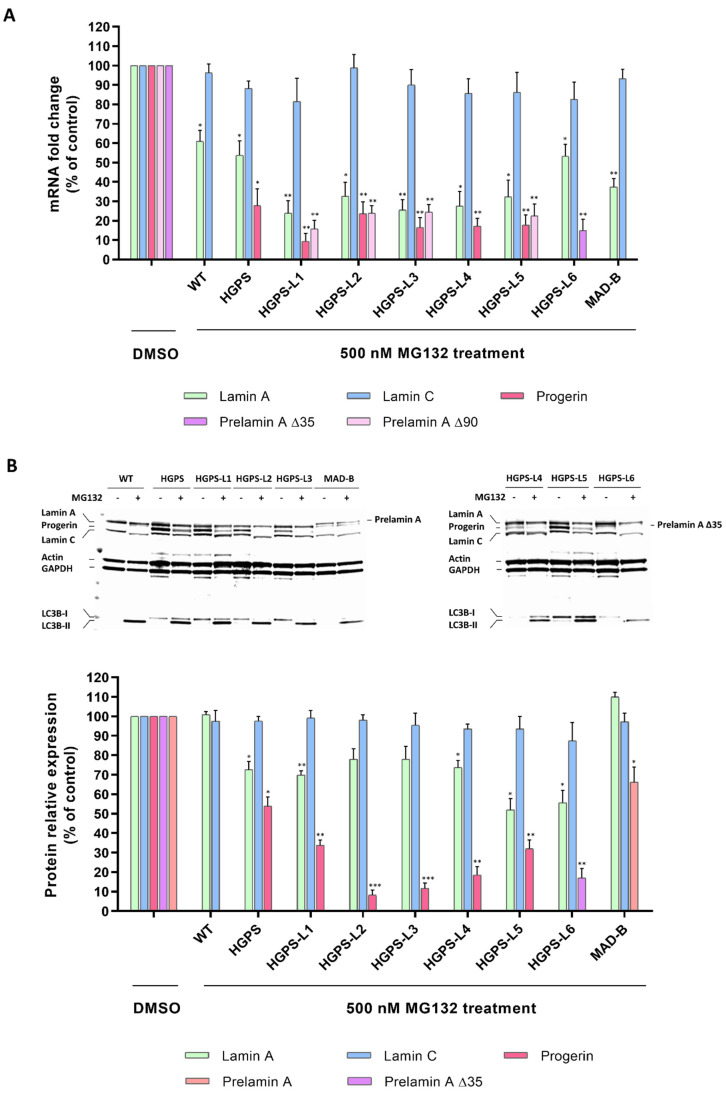
MG132 reduces aberrant prelamin A levels in HGPS-like and MAD-B fibroblasts. (**A**) Downregulation of aberrant prelamin A transcripts (Δ50: progerin, Δ35, Δ90, and WT prelamin A) in response to MG132. Quantitative real-time PCR analyses of lamin A, prelamin A Δ50 (progerin), prelamin A Δ35, prelamin A Δ90, lamin C, and RPS13 mRNA levels in HGPS, HGPS-like, MAD-B, and WT fibroblasts treated for 24 h with 500 nM MG132 relative to DMSO-treated cells (Control). The fold change of each transcript was determined by normalizing its value to that of RPS13 for each condition. (mean ± SEM, *n* = 4, Student’s *t*-test, * *p* < 0.05, ** *p* < 0.01, experimental vs. control). (**B**) MG132 reduced aberrant prelamin A protein levels in HGPS-like and MAB-B patient cells. Upper panels: Western blotting evaluation of lamin A/C, progerin, prelamin A, prelamin A ∆35 in whole-cell lysates from WT, HGPS, HGPS-like, and MAD-B fibroblasts treated with DMSO (−), 500 nM MG132 for 48 h (+). Lower panels: lamin A/C, progerin, prelamin A, prelamin A ∆35 expression levels were normalized to GAPDH values using ImageJ software. (mean ± SEM, *n* = 3, Student’s *t*-test, * *p* < 0.05, ** *p* < 0.01, *** *p* < 0.001. MG132-treated vs. DMSO-treated cells).

Our previous studies in typical HGPS cells have shown that MG132 promotes both progerin degradation through autophagy activation and reduction of progerin synthesis mediated by the regulation of SRSF-1 and SRSF-5, playing the opposite role in the utilization of the *LMNA* and progerin 5′ splice site. Therefore, hypothesizing that MG132 might have the same effects on aberrant prelamin A isoforms clearance in HGPS-like cells, we performed quantitative reverse transcription–polymerase chain reaction (RT-PCR) assays using primers specific for prelamin A mRNA isoforms in MG132- and DMSO-treated HGPS-like cells. As shown in Figure 2A, when compared to DMSO-treated cells, MG132 treatment at 500 nM for 24 h induces aberrant prelamin A mRNA downregulation, suggesting that the drug acts at the transcriptional levels. Indeed, we observed significant reductions in prelamin A Δ50 and prelamin A Δ90 mRNAs in HGPS-L1, HGPS-L2, HGPS-L3, and HGPS-L5 patients’ cells, prelamin A Δ50 mRNA in HGPS-L4 patients’ cells, and prelamin A Δ35 mRNA in HGPS-L6 patients’ cells. The treatment also significantly decreased the production of lamin A transcripts in MAD-B fibroblasts.

To further evaluate the MG132-associated decrease in prelamin A isoforms at the protein levels, we treated HGPS-like fibroblasts with 500 nM MG132 for 48 h. Quantification of the Western blotting experiments revealed clear reductions in prelamin A Δ50 in HGPS-L1, HGPS-L2, HGPS-L3, HGPS-4, and HGPS-L5 patients’ cells and prelamin A Δ35 in HGPS-L6 patients’ cells. In MAD-B cells, the treatment also significantly decreased the production of prelamin A (Figure 2B). Interestingly, in all the tested HGPS-like cell lines and concomitantly with the decrease of aberrant prelamin A levels, the LC3B-I to LC3B-II autophagic switch was increased.

### 3.3. MG132 Reduces Senescence, Enhances Proliferation and Migration in HGPS-like and MAD-B Patient Cells

**Figure 3:**
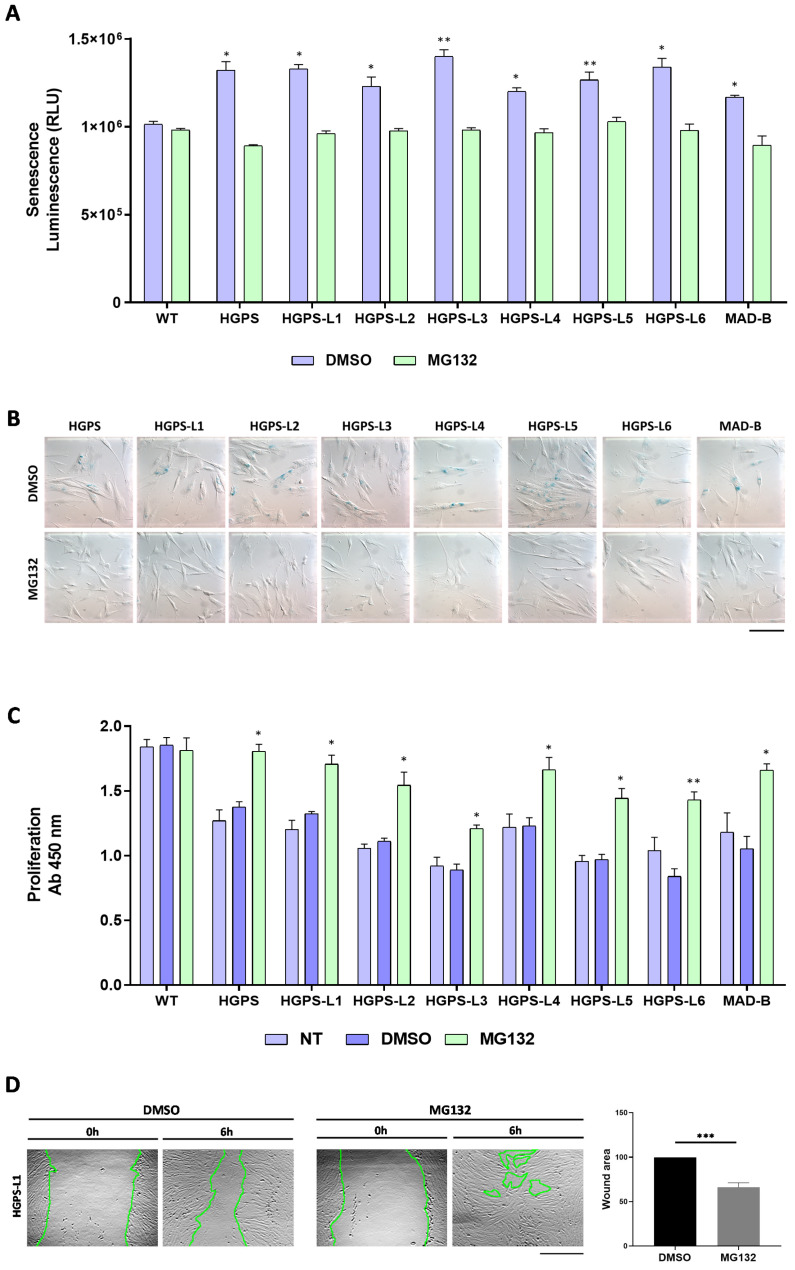
MG132 reduces senescence and enhances proliferation and migration in HGPS-like and MAD-B patients’ cells. (**A**) Luminescence-based quantification of senescence rate in WT, HGPS-like, and MAD-B fibroblasts treated with 500 nM MG132 for 96 h relative to DMSO-treated cells. Each experiment was performed on cells at the same passage level. Senescence is determined as relative light units (RLUs). (mean ± SEM, *n* = 3, Student’s *t*-test, * *p* < 0.05, ** *p* < 0.01. MG132-treated vs. DMSO-treated cells). (**B**) Colorimetric detection of senescence-associated β galactosidase in HGPS-like and MAD-B fibroblasts treated with 500 nM MG132 for 96 h relative to DMSO-treated cells. Each experiment was performed on cells at the same passage level. β-galactosidase blue staining is lower in cells treated with MG132 compared to cells treated with DMSO. (**C**) Cell proliferation rate based on the incorporation of bromodeoxyuridine (BrdU) into the DNA was expressed as absorbance OD 450 nm. (**D**) Left panel: an example of wound healing assay performed on HGPS-L1 fibroblasts treated for 6 h with DMSO or MG132 (500 nM). Right panel: the results of wound healing assays on individual samples (Figure S1) were grouped into biological replicates (1 HGPS, 6 HGPS-like, and 1 MAD-B) to perform statistical tests. (mean ± SEM, *n* = 8, Student’s *t*-test, *** *p* < 0.001. MG132-treated vs. DMSO-treated cells). Results are expressed as a percentage of the area of the original wound and normalized to DMSO-treated cells, considered as 100%. Scale bar, 100 µm.

In primary fibroblasts from HGPS patients, progerin accumulation results in premature senescence, a major hallmark of HGPS, as well as of normal aging cells [19,20,21]. Therefore, we hypothesized that MG132-induced clearance of progerin might delay senescence in HGPS-like cells. To test MG132 efficacy, we first measured senescence by quantification of a luminescent signal that is dependent on and correlates with β-galactosidase activity. Interestingly, all HGPS-like cells treated with 500 nM MG132 for 96 h exhibited a decreased senescence rate (Figure 3A). Furthermore, using Senescence Associated β-galactosidase staining, we observed that this MG132 treatment scheme induces a decrease in the number of senescent cells when compared to DMSO-treated cells (Figure 3B).

Primary fibroblasts from HGPS patients exhibit proliferative defects [19]. To determine whether MG132-induced clearance of progerin has any beneficial effects on cell proliferation, we examined the proliferation rates of HGPS-like fibroblasts with MG132 or DMSO treatment and found that in all the tested cell lines, proliferation rates were increased by a 96 h MG132 treatment at 500 nM when compared to the DMSO-treated cells (Figure 3C). As in HGPS [22], nuclear architecture and cell migration are impaired during physiological aging [23]. We investigated the effect of MG132 treatment on cell migration. “Wound-healing” assays showed that most of the MG132-treated HGPS-like and MAD-B cells (6/8) were able to migrate and to “heal the wounds” better than their control DMSO-treated counterparts (Figure 3D and Figure S1).

### 3.4. MG132 Treatment Rescues the Level of Proteins Whose Expression Is Altered in HGPS-like and MAD-B Cells

**Figure 4:**
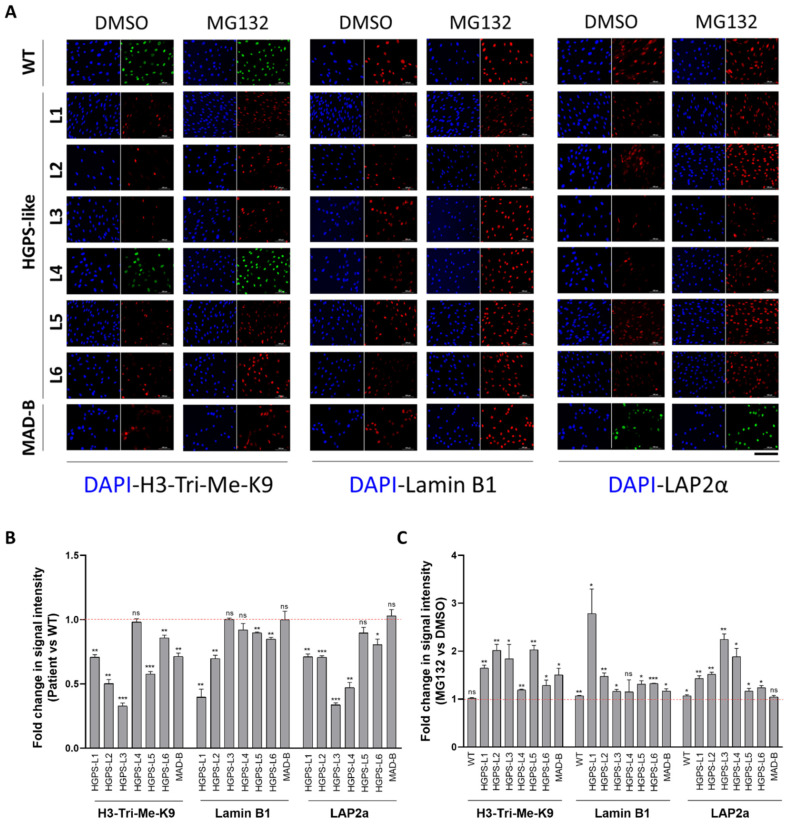
MG132 treatment rescues the level of proteins, the expression of which is altered in HGPS-like and MAD-B cells. (**A**) Immunofluorescence microscopy on primary dermal fibroblasts from a healthy individual (WT) and HGPS-like and MAD-B patients, treated with 500 nM MG132 or an equal volume of DMSO for 48 h. Cells were stained with DAPI (blue) and antibodies to tri-methyl lysine 9 of core histone H3 (H3-Tri-Me-k9), lamin B1, and LAP2α. (**B**) Fold change in signal intensity in DMSO-treated patient’s cells relative to DMSO-treated WT cells, each normalized to the corresponding nuclei number. The signal intensity was set to 1 in DMSO-treated WT cells (mean ± SEM, *n* = 3, Student’s *t*-test, * *p* < 0.05, ** *p* < 0.01, *** *p* < 0.001). (**C**) Fold change in signal intensity in MG132-treated relative to DMSO-treated HGPS fibroblasts, each normalized to the corresponding nuclei number. The signal intensity was set to 1 in DMSO-treated cells. At least 200 fibroblast nuclei were randomly selected for each cell line (mean ± SEM, *n* = 3, Student’s *t*-test, * *p* < 0.05, ** *p* < 0.01, *** *p* < 0.001) and examined using ImageJ software. Scale bar, 200 µm.

Other characteristics of fibroblasts from individuals with HGPS cells include a loss of peripheral heterochromatin and downregulated tri-methyl lysine 9 of core histone H3 (H3-Tri-Me-K9) [20,21] as well as reduced levels of the nuclear components, lamin B1, and lamina-associated polypeptide (LAP2α) [19,20]. By immunocytochemistry studies, as observed in Figure 4A and Figure S2 (larger images), levels of these proteins are also reduced in HGPS-like and MAD-B cells compared to WT (Figure 4B), supporting a negative correlation between aberrant prelamin A accumulation and the downregulation of several nuclear proteins, including histone modification patterns. Importantly, treatment with MG132 restored the levels of histone H3-Tri-Me-K9, lamin B1, and LAP2α in most cells (Figure 4C).

### 3.5. Treatment of HGPS-like and MAD-B Cells with MG132 Reduces the Levels of DNA Damage

**Figure 5:**
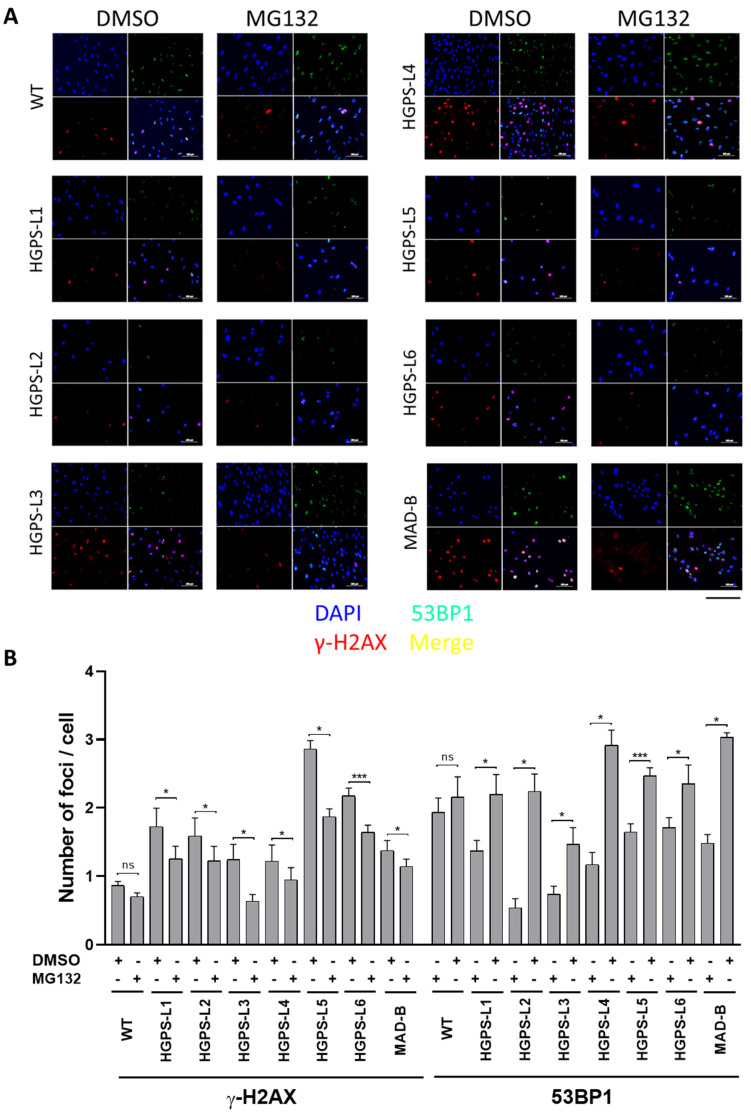
Treatment of HGPS-like and MAD-B cells with MG132 reduces the levels of DNA damage. (**A**) Immunofluorescence microscopy on primary dermal fibroblasts from a healthy individual (WT) and HGPS-like and MAD-B patients, treated with 500 nM MG132 or an equal volume of DMSO for 48 h. Cells were stained with DAPI (blue) and antibodies to the indicated proteins. Scale bar, 200 µm. (**B**) Quantification of the number of foci of γ-H2AX and 53BP1 per cell in MG132-treated fibroblasts compared to DMSO-treated fibroblasts (mean ± SEM, *n* = 3, Student’s *t*-test, * *p* < 0.05, *** *p* < 0.001).

Previous studies have shown that HGPS cells accumulate a defective DNA damage response (DDR), playing a key role in the premature aging phenotypes [24,25]. Progerin causes chromatin perturbations, especially the global loss of histone H3-Tri-Me-K9, leading to the formation of DSBs (double-strand breaks) and abnormal DDR, as evidenced by the accumulation of phosphorylated histone γ-H2AX foci and the impaired recruitment of p53-binding protein 1 (53BP1) to sites of DNA damage [26,27]. We performed γ-H2AX/53BP1 double immunofluorescence staining and observed more γ-H2AX-positive foci in HGPS-like and MAD-B cells than those observed in control, with defective recruitment of 53BP1 to these sites (Figure 5A and Figure S3: larger images). However, MG132 treatment reduced the number of nuclei with γ-H2AX foci. Moreover, we observed a more effective recruitment of 53BP1 to the remaining γ-H2AX foci (Figure 5B).

### 3.6. Anti-Inflammatory Effects of MG132 in HGPS-like and MAD-B Cells

Many altered signaling pathways have been described in HGPS cells [10,21], among them, the hyperactivation of the NF-κB inflammatory pathway [28]. In a previous study, crossing a mouse model for premature aging, Zmpste24−/−, with transgenic mice displaying reduced NF-κB signaling, extended longevity, and prevented the development of progeroid features. Moreover, the inhibition of NF-κB by sodium salicylate efficiently prevented the disease phenotypes in Zmpste24-deficient mice and extended longevity in the HGPS mouse model, *Lmna*^G609G/G609G^ [28]. On the other hand, MG132 is also known to attenuate the degradation of NF-κB inhibitor, I-κB (Figure S4), resulting in the inhibition of proinflammatory cytokine secretion [29,30,31].

**Figure 6:**
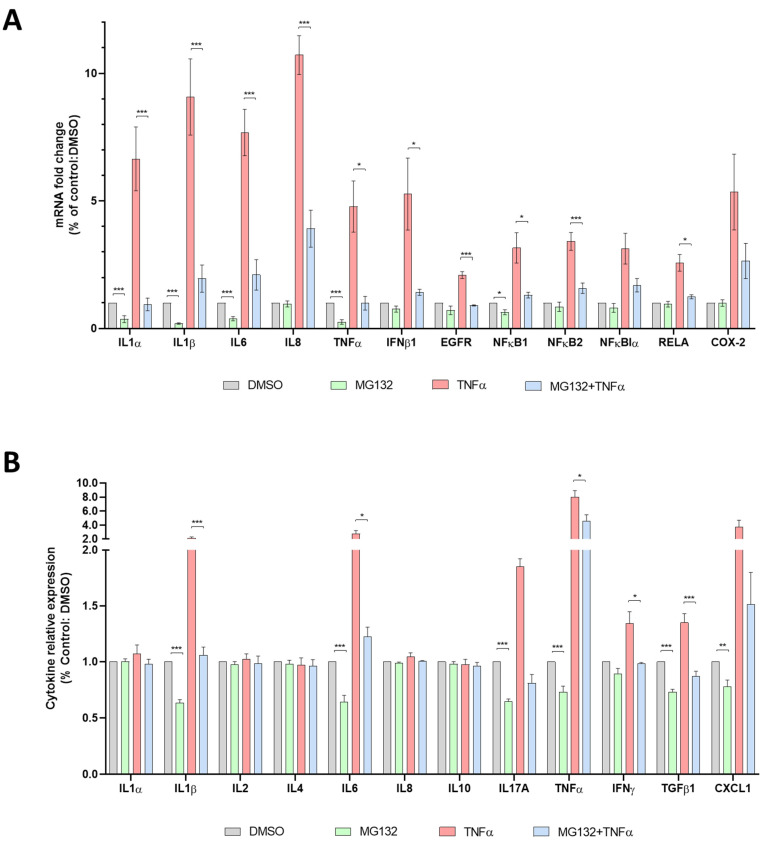
Anti-inflammatory effects of MG132 in HGPS-like and MAD-B cells. (**A**) Quantitative real-time PCR using selected inflammatory genes expression arrays in culture supernatants of HGPS, HGPS-like, and MAD-B fibroblasts treated for 6 h with MG132 (500 nM), TNFα (10 ng/mL) alone and in combination, or DMSO as vehicle control. The results of individual samples (Figure S5) were grouped into biological replicates (1 HGPS, 6 HGPS-like, and 1 MAD-B) to perform statistical tests. (mean ± SEM, *n* = 8, Student’s *t*-test, * *p* < 0.05, *** *p* < 0.001; MG132-treated vs. DMSO-treated cells and MG132+TNFα-treated vs. MG132-treated cells). (**B**) Enzyme-linked immunosorbent assay (ELISA) using multi-analyte ELISA arrays to measure inflammatory cytokines in culture supernatants from HGPS, HGPS-like, and MAD-B fibroblasts treated for 24 h with MG132 (500 nM), TNFα (10 ng/mL) alone and in combination, or DMSO as vehicle control. The results of individual samples (Figure S6) were grouped into biological replicates (1 HGPS, 6 HGPS-like, and 1 MAD-B) to perform statistical tests. (mean ± SEM, *n* = 8, Student’s *t*-test, * *p* < 0.05, ** *p* < 0.01, *** *p* < 0.001; MG132-treated vs. DMSO-treated cells and MG132+TNFα-treated vs. MG132-treated cells).

To further investigate the cellular inflammatory response of MG132-treated HGPS fibroblasts, and given that this cell type (fibroblasts from skin biopsy) is known to secrete high levels of inflammatory cytokines [32], we performed RNA-seq experiments (accession number: E-MTAB-5807) and analyzed the expression levels of NF-κB gene signatures in classical HGPS fibroblasts treated with MG132. Interestingly, we found a decrease in the transcript’s levels of TNFα, IL-6, IL-18, IL-19, IL-34, IL-1 receptor accessory, IFNα-R2, interferon regulatory factor 7, TGFβ-R3, and EGF-R, as well as the increase of other anti-inflammatory transcripts: IL-1R2, IL-1R antagonist, NF-κB inhibitor α, NFκB inhibitor β, NFκB repressing factor, and NF-κB inhibitor like 1 (Figure S5). In order to assess the inflammatory response with MG132 on HGPS-like and MAD-B fibroblasts, we performed quantitative real-time PCR using selected inflammatory genes expression arrays on culture supernatants of fibroblasts treated with MG132, TNFα alone, and in combination. As described in Figure 6A and Figure S6, we found that MG132 reduces the transcript levels of proinflammatory cytokines (IL-1α, IL-1β, IL-6, TNFα) in HGPS-like and MAD-B patient cells. Moreover, treatment with MG132 reduced the transcript levels of proinflammatory mediators induced by recombinant TNFα (IL-1α, IL1-β, IL-6, IL-8, TNFα, IFNβ1, EGF-R, NFκB1, NFκB2, RelA). In the same way, using ELISA, we found a significant downregulation of several proinflammatory cytokines, such as IL-1β, Il-6, IL-17A, TNFα, TGFβ, and CXCL1. Again, MG132 reduces the TNFα-induced secretion of the proinflammatory cytokines IL-1β, Il-6, TNFα, IFNγ, and TGFβ (Figure 6B and Figure S7).

## 4. Discussion

We previously showed that the benefit of MG132 on classical HGPS fibroblasts and mice is mediated by 1/induced macroautophagy, leading to progerin degradation and 2/blocking progerin production by reducing SRSF-1 expression levels and increasing expression levels of SRSF-5, controlling the aberrant splicing of prelamin A precursor mRNA. MG132 treatment improves HGPS fibroblast phenotypes, reduces cell senescence, and improves their viability and proliferation. Injection of MG132 into the skeletal muscle of our progeria mice model (*Lmna*^G609G/G609G^) locally reduced progerin and SRSF-1 expression levels [14].

*LMNA* mutations other than the classical c.1824C>T (p.G608G) have been shown to cause the production of progerin and/or other truncated or wild-type prelamin A isoforms in patients affected with HGPS-like and MAD-B syndromes [15,33]. In HGPS and HGPS-like syndromes, aberrant prelamin A isoforms lack the C-terminal ZMPSTE24 cleavage site and retain their farnesyl group. The mutated proteins remain anchored to the inner nuclear envelope within the nuclear lamina. This localization impairs several biological parameters, leading to accelerated aging. Besides characteristic nuclear envelope deformations, progerin disrupts nucleo-cytoplasmic exchanges through alterations in the nuclear pore complex composition and in the expression of factors involved in protein transport [34,35]. Moreover, progerin sequesters, within the nuclear lamina, several proteins that control cell proliferation, DNA repair, signaling pathways, and metabolic responses such as NRF2, regulating oxidative stress [36]. Among the deregulated signaling pathways, an enhanced NF-κB activity is responsible for the inflammatory signaling and the senescence-associated secretory phenotype [37]. Farnesylated progerin has also been shown to impair nuclear mechanical characteristics, DNA damage responses, chromatin organization, telomere length, gene expression, mitosis, stem cell exhaustion, and the deregulation of extracellular matrix production and remodeling [21,38,39].

Several strategies have been developed to correct some of these abnormalities, either in cell cultures, in animal models, or in progeria patients, targeting progerin farnesylation, clearance, abnormal splicing, or downstream effects. MG132 has been shown to enhance progerin clearance in human cell cultures and in the skeletal muscle of *Lmna*^G609G/G609G^ progeria mice model, thus correcting some biological parameters. We, therefore, hypothesized that MG132 could also have a beneficial impact on HGSP-like and MAD-B cells since they share the same pathophysiological mechanism based on the abnormal splicing of prelamin A pre-mRNA. To this end, we evaluated the treatment’s efficacy of MG132 in reducing the production of all prelamin A isoforms, including aberrantly accumulated prelamin A, either truncated (HGPS-like) or wild type (MAD-B). Here, we show a significant decrease of each aberrant transcript’s production (prelamin A ∆35, ∆50, and ∆90) as well as the corresponding abnormal proteins. Interestingly, MG132 not only induces the synthesis blockade of the aberrant prelamin A isoforms and the clearance of the corresponding proteins already expressed but also results in the improvement of several biological parameters, including cellular senescence, proliferation, altered protein expression, DNA damage and repair, as well as inflammatory cytokine expression. Given that the accumulation of aberrant prelamin A isoforms and, consequently, the alteration of WT lamin A functions cause several side effects, it is not surprising that the clearance of these aberrant isoforms improves the phenotype of the patients’ cells.

**Figure 7:**
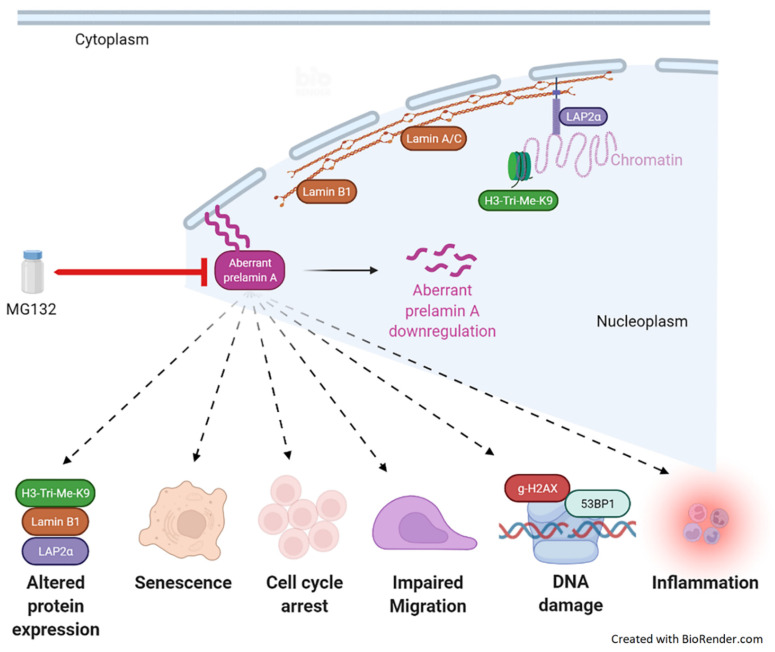
Schema summarizing the results of the current study. MG132 reduces the transcript levels of the aberrant prelamin A isoforms and the corresponding proteins already expressed. MG132 treatment improves several pathological parameters, including altered protein expression, cellular senescence, cell cycle arrest, impaired migration, DNA damage, as well as inflammatory cytokine expression.

The results of the present study, summarized in Figure 7, demonstrate that MG132 is at least as efficient as morpholinos treatment [16], with a wide range of beneficial effects on HGPS and HGPS-like, potentially due to both an impressive clearance of aberrant prelamin A isoforms and the rescue of downstream noxious cascades. Notably, MG132 treatment lowers the levels of mediators of the inflammatory pathways. In agreement with our study, MG132 is known to block the degradation of the NF-κB inhibitor (I-κB) to inhibit the secretion of proinflammatory cytokines, resulting in the abolition of NF-kB activation in several cell types, including the human myeloid leukemia cell line U937 [29], dental pulp stem cells [30], mice gastrocnemius muscles [40], or the rat renal tubular epithelial cell line [41]. Inflammation is a major regulator of the physiological and premature aging process [42]. Moreover, the major clinical hallmark of progeria is atherosclerosis, leading to premature death by myocardial infarction or stroke [1,40,41]. These findings, together with the fact that arterial lesions in both typical atherosclerosis and HGPS exhibit inflammation, calcification, and the loss of vascular smooth muscle cells (VSMCs) [43,44], support the need of targeting the inflammation signaling cascade for the treatment of premature aging disorders.

On the other hand, the NRF2 antioxidant pathway has been described as a driver mechanism in HGPS due to impaired NRF2 transcriptional activity and, consequently, increased chronic oxidative stress [45]. Importantly, it has been shown that MG132 activates the NRF2-ARE signaling pathway, which is associated with increased Nrf2 transcription and expression, leading to the prevention of oxidative stress, induced both in cardiovascular and renal injury [46] and in several human endothelial and vascular smooth muscle cells [47,48]. Furthermore, MG132 was reported to have a significant preventive and therapeutic effect on accelerated atherosclerosis in rabbits [49], diabetic cardiomyopathy in a diabetic mouse model [50], and arthritis associated with joint inflammation in rats [51]. Interestingly, these features are exhibited by HGPS patients who might benefit from the same treatment.

In the present study, we showed that MG132 treatment improves the migration of most cell lines. Matrix metalloproteinases (MMPs) could influence the wound healing parameter. Indeed, it has been shown that MMP-3 messenger RNA and protein levels decreased significantly in HGPS fibroblasts [52]. MMP3 degrades extracellular matrix proteins, such as collagen types II, IV, and IX, and activates other MMPs, such as MMP1, -7, and -9. MMP3 has also been shown to facilitate cellular migration and invasion [53,54]. Interestingly, Bortezomib (a proteasome inhibitor used in humans as a therapeutic agent for multiple myeloma) has been shown to elicit an anti-fibrosis effect through a dual activity: an increase in MMP1 and MMP2 mRNA and proteins and a decrease in collagen 1a mRNAs and proteins [55]. We thus hypothesize that the MG132 effect on cell migration could be mediated by the MMPs’ protein modulation.

In order to evaluate the effects of MG132 in our Knock-in progeria mouse model (*Lmna*^G609G/G609G^), carrying the c.1827C>T (p.Gly609Gly) mutation, we previously showed that the reduction of progerin levels upon IV or IP systemic treatment was not significant, suggesting that the molecule is unstable when injected systemically. Therefore, we performed intramuscular injections in *Lmna*^G609G/G609G^ tibialis anterior muscle. In this case, treatment with MG132 induced a significant decrease of progerin and SRSF-1 levels in the treated muscle compared to the untreated contralateral muscle [14].

MG132 rapid catabolism upon IV or IP administration is a clear limiting step for the systemic delivery of the drug. We, therefore, set up a collaboration with an academic laboratory to develop MG132-derivatives in order to optimize the chemistry of the molecule, to improve its stability and efficacy, and to analyze and minimize adverse effects, aiming to obtain in vivo systemic efficacy on the reversion of premature aging phenotypes in *Lmna*^G609G/G609G^ mice.

Altogether, the originality and therapeutic potential of MG132 for HGPS and related diseases is based on its triple mechanism of action: targeting progerin production and degradation, in combination with decreased downstream noxious effects. Here, we have provided evidence that the use of MG132 could be extended to other syndromes characterized by the accumulation of truncated or wild-type prelamin A. Our results establish a preclinical proof of principle for the use of MG132 or its druggable derivatives in HGPS-like and MAD-B syndromes, with a strong potential for clinical administration in future trials.

## Figures and Tables

**Table 1:** List of genes used in real-time PCR using inventoried TaqMan Gene Expression Arrays.

Gene	ID
18s rRNA	Hs99999901_s1
IL-1α	Hs00174092_m1
IL-1β	Hs01555410_m1
IL-6	Hs00174131_m1
IL-8	Hs00174103_m1
TNFα	Hs00174128_m1
IFN-β1	Hs01077958_s1
EGFR	Hs01076090_m1
NFκB1	Hs00765730_m1
NFκB2	Hs01028890_g1
NFκBIα	Hs00355671_g1
RELA	Hs01042014_m1
COX-2	Hs00153133_m1

## Data Availability

The authors state that all data generated during this study are included in the article and that they are available from the corresponding author upon reasonable request. RNA seq data that support the findings of this study have been deposited in ArrayExpress under the accession number E-MTAB-5807 (Harhouri; 2017-06-1; RNA seq of HGPS treated cells; ArrayExpress; E-MTAB-5807).

## Supplementary Materials

The following supporting information can be downloaded at: https://www.mdpi.com/article/10.3390/cells11040610/s1, Figure S1: MG132 promotes HGPS-like and MAD-B fibroblasts migration, Figure S2: Larger images of Figure 4, Figure S3: Larger images of Figure 5, Figure S4: MG132 blocks the degradation of NF-κB inhibitor, I-κB, Figure S5: Transcriptional attenuation of inflammatory response to MG132 in classical HGPS fibroblasts, Figure S6: MG132 reduces the transcript levels of proinflammatory mediators and counteracts TNFα-induced inflammation, Figure S7: MG132 reduces the secretion of proinflammatory cytokines and alleviates TNFα-induced inflammation.
