# Supplementary material for: MG132 Induces Progerin Clearance and Improves Disease Phenotypes in HGPS-like Patients’ Cells
Source: Cells. 2022 Feb 10;11(4):610. doi: 10.3390/cells11040610 (PMC8870437; doi:10.3390/cells11040610)
Supplement: Supplementary file 1 [file cells-11-00610-s001.zip › cells-1567427-supplementary.pdf]

cells MDPI

Supplementary Materials:

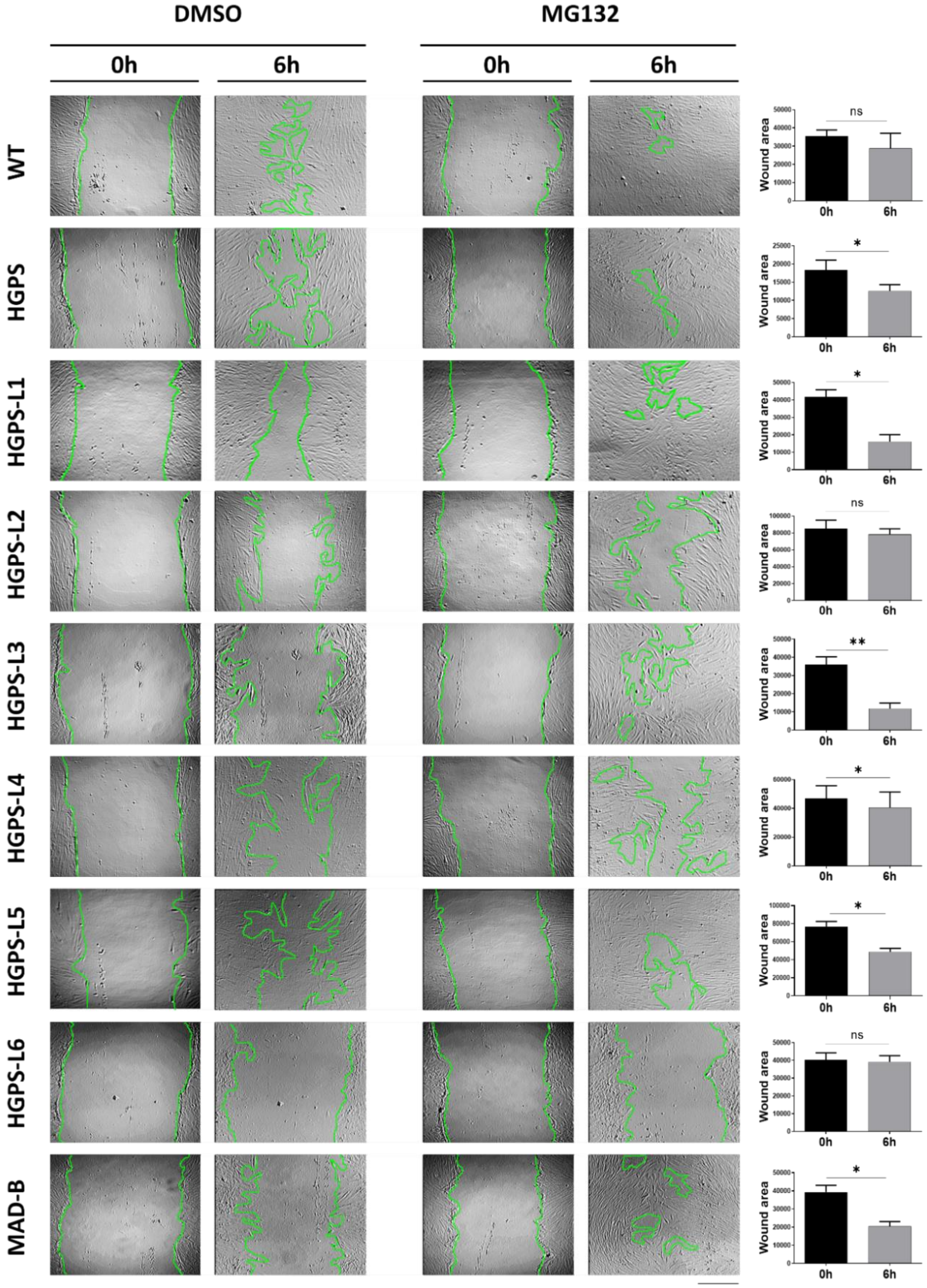

Figure S1. MG132 promotes HGPS-like and MAD-B fibroblasts migration**.** Wound healing assay performed on WT, HGPS, HGPS-like and MAD-B fibroblasts treated for 6h with DMSO or MG132 (500 nM). After subtracting the DMSO-induced wound repair during 6 h (control), results were expressed as the wound area following MG132 treatment (6 h) compared to the original wound (0 h). (mean ± SEM, n = 3, Student's t-test, * $p < 0.05$, ** $p < 0.01$). Scale bar, 100 µm.

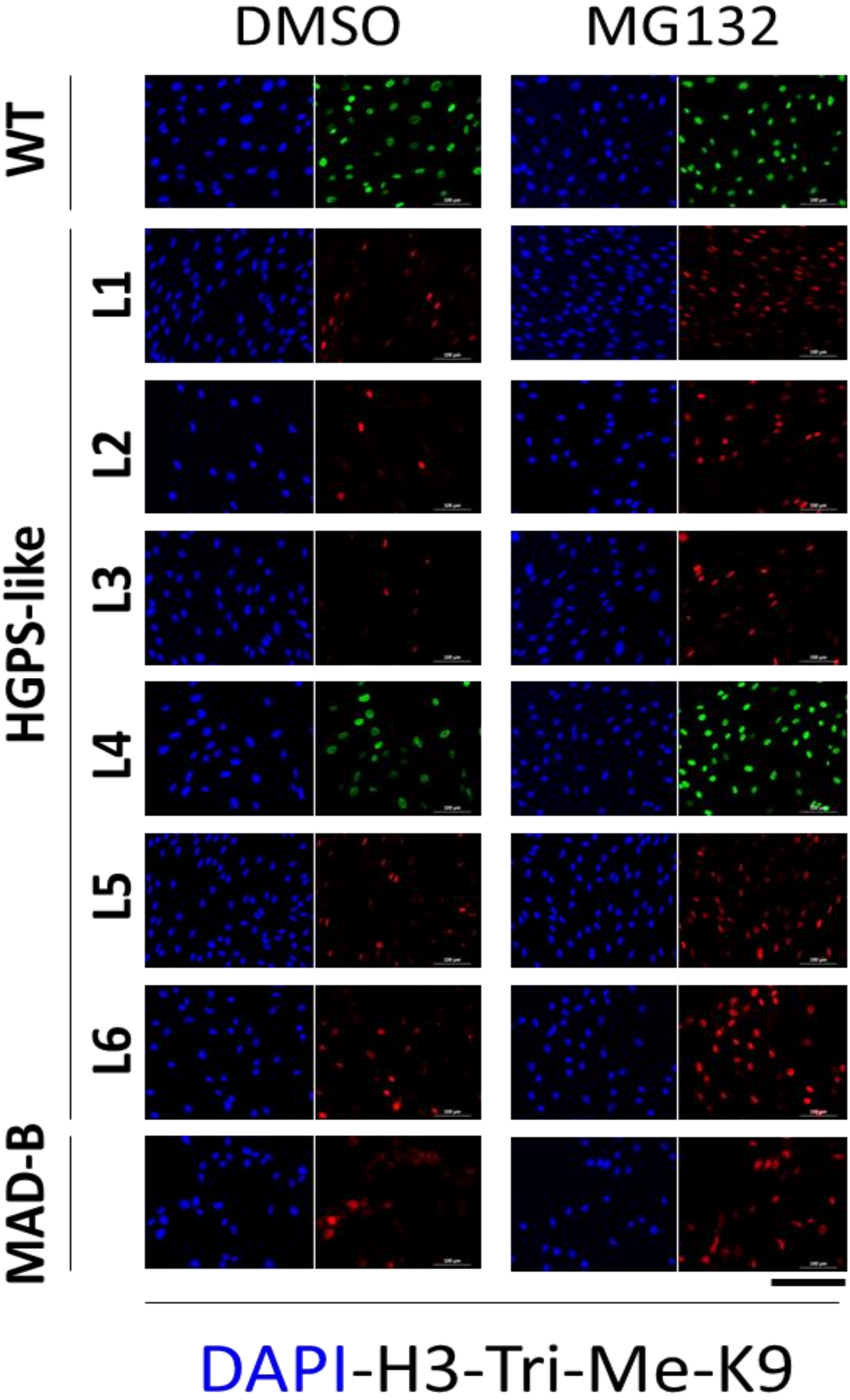
DMSO
MG132
WT
HGPS-like
L1
L2
L3
L4
L5
L6
MAD-B
DAPI-H3-Tri-Me-K9

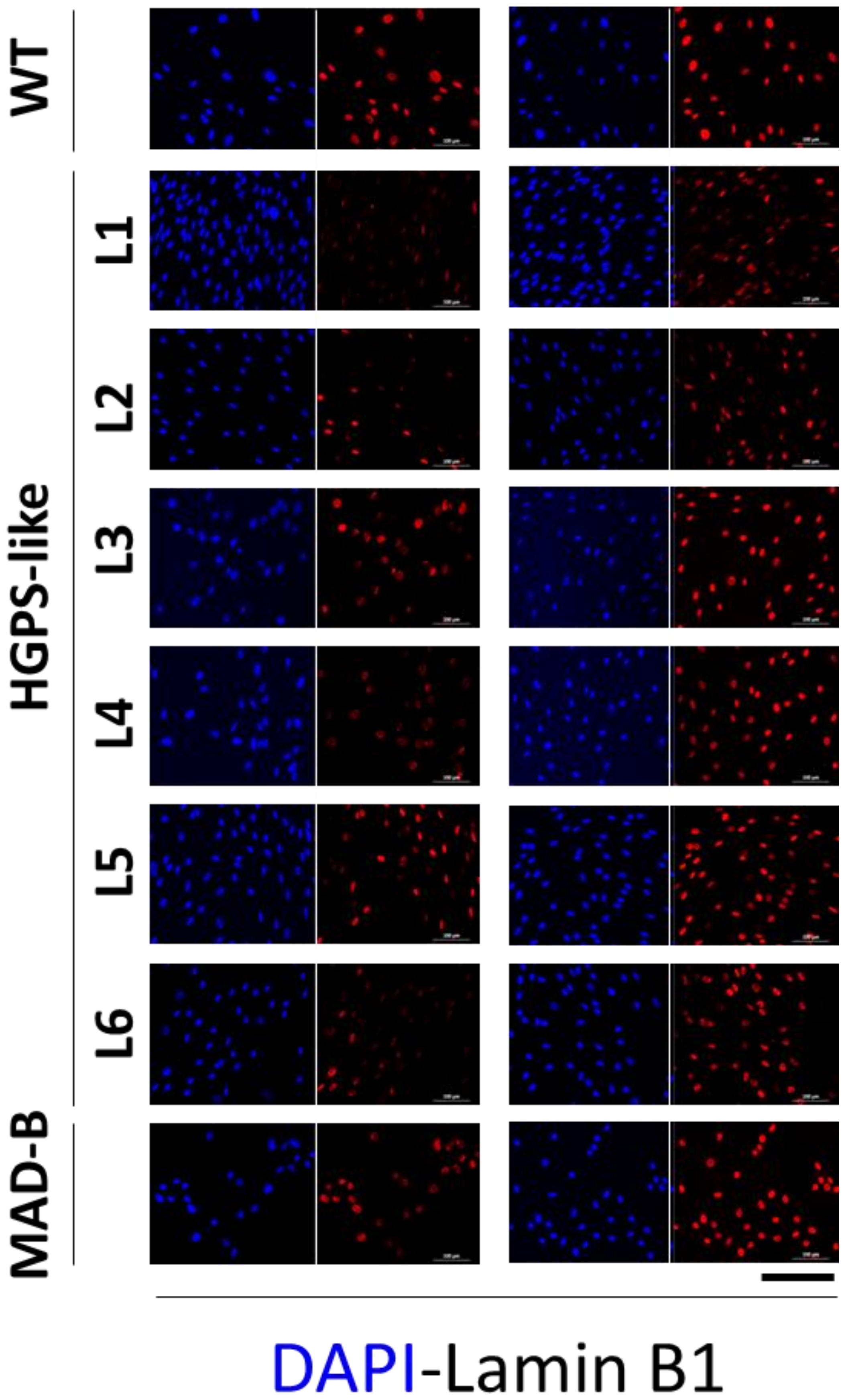
WT
HGPS-like
L1
L2
L3
L4
L5
L6
MAD-B
DAPI-Lamin B1

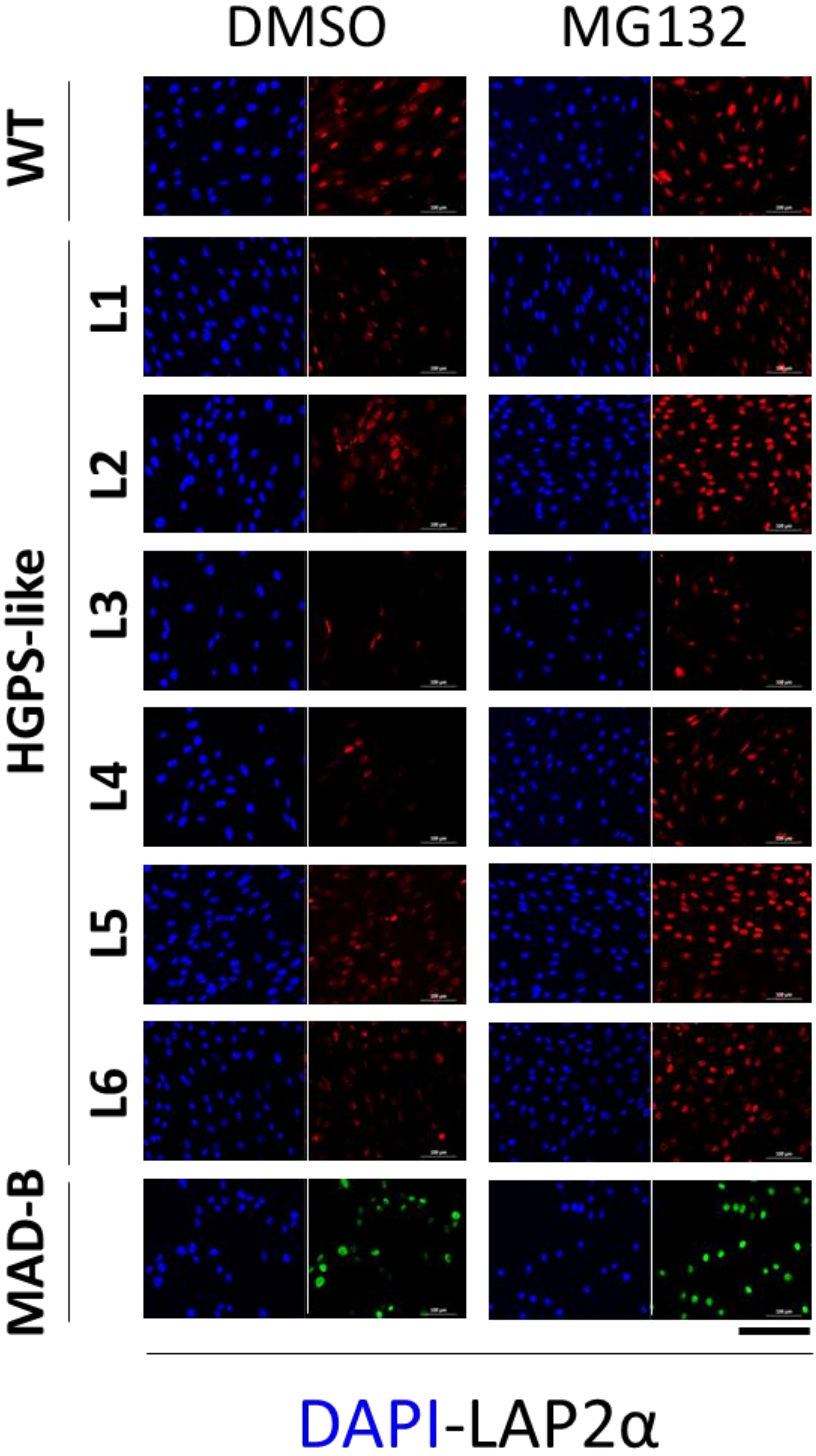

Figure S2. Larger images of Figure 4

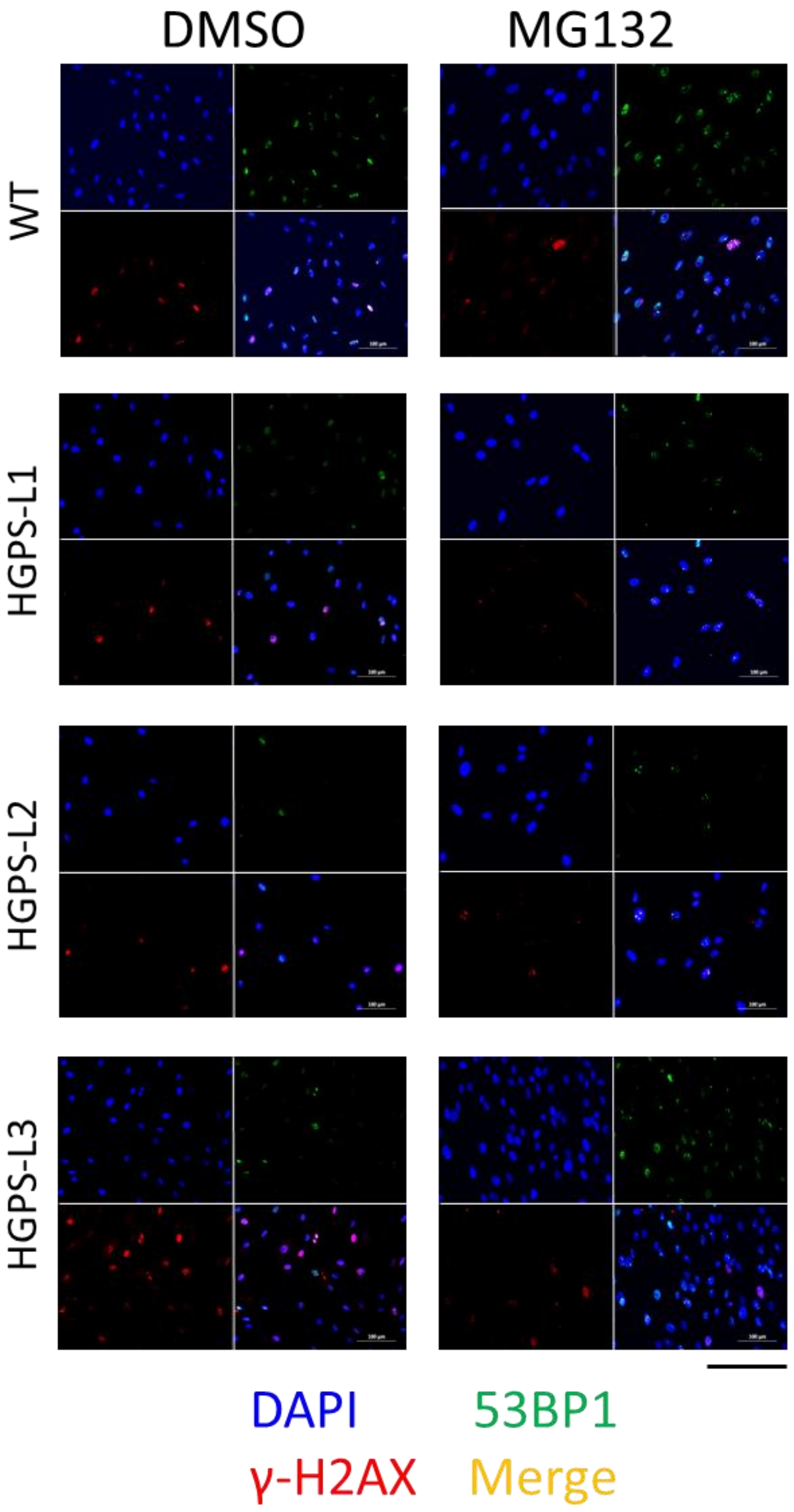
DMSO
MG132
WT
HGPS-L1
HGPS-L2
HGPS-L3
DAPI
53BP1
γ-H2AX
Merge

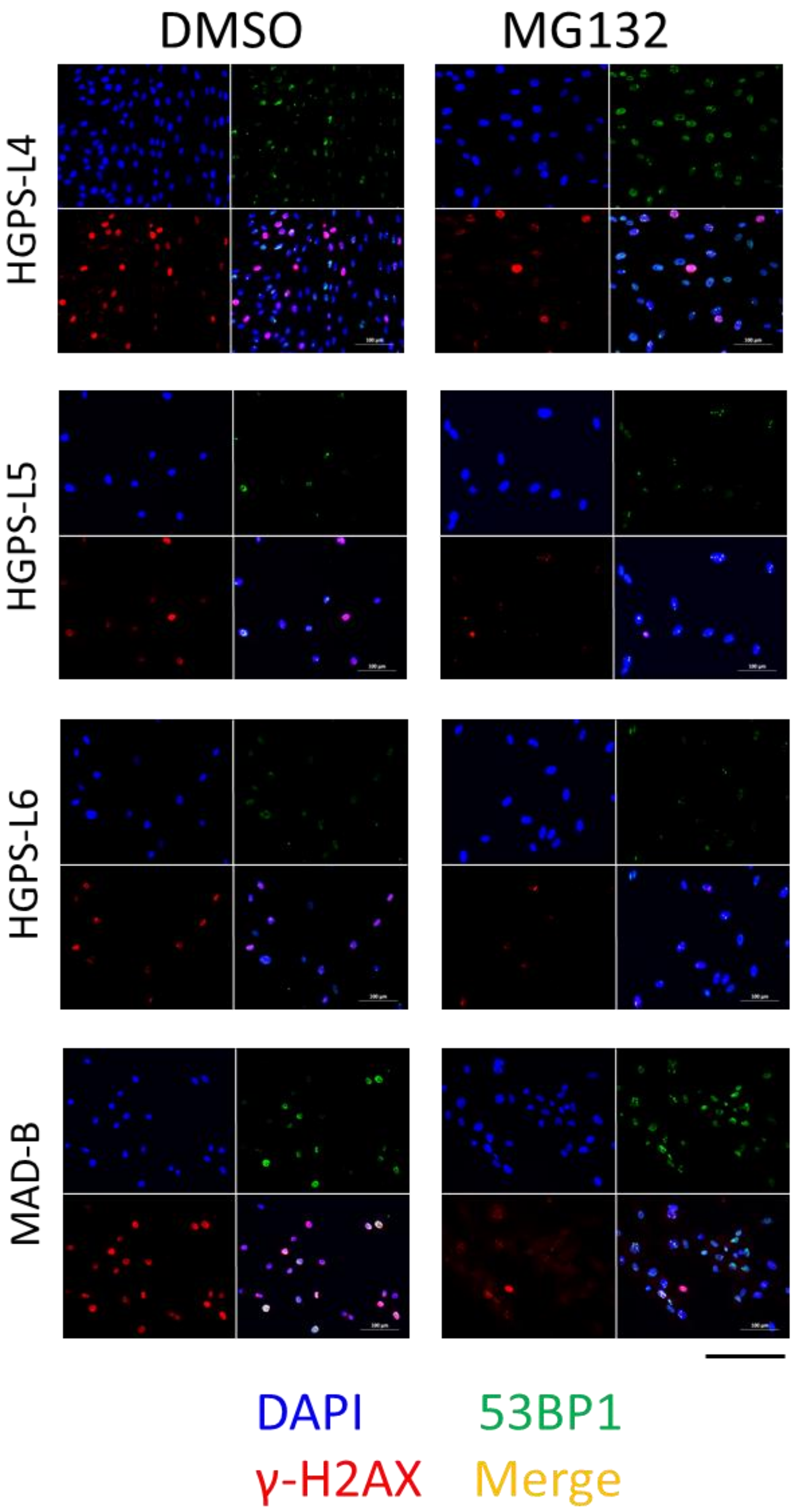

Figure S3. Larger images of Figure 5

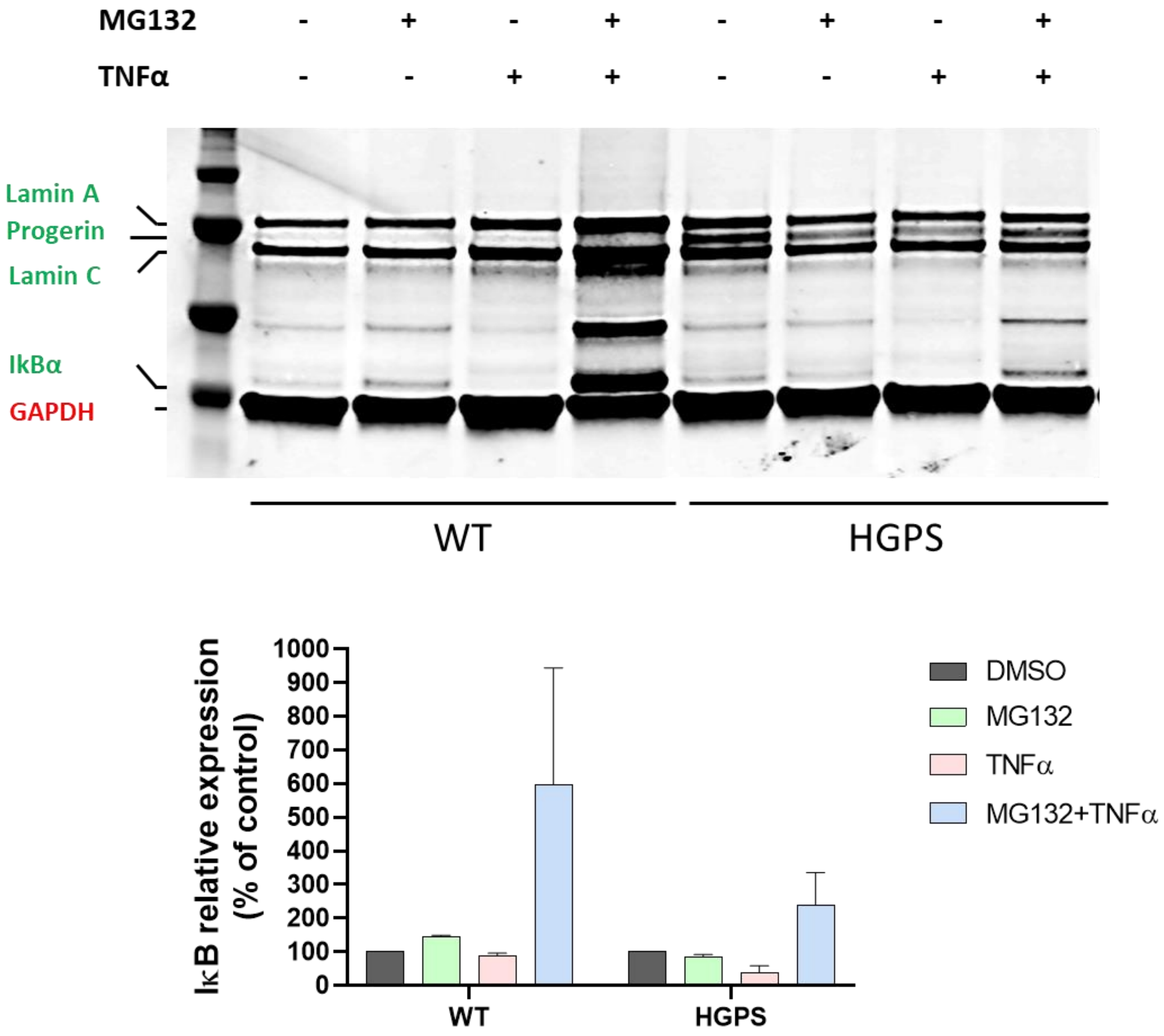

Figure S4. MG132 blocks the degradation of NF-κB inhibitor, I-κB. Upper panel: Western blotting evaluation of Lamin A/C and I-κB in whole cell lysates from WT and HGPS fibroblasts treated with DMSO (-), 500 nM MG132 for 48 h (+), 10 ng/ml TNFα for 48 h (+) alone or in combination. Lower panel: I-κB expression levels were normalized to GAPDH values using ImageJ software.

Description	Gene_position	Gene	Fold change
tumor necrosis factor	chr6:31575567-31578336	TNFα	0.55
interleukin 1 beta	chr2:112829751-112836903	IL1β	0.98
interleukin 6	chr7:22725884-22732002	IL6	0.58
interleukin 18	chr11:112143251-112164117	IL18	0.46
interleukin 19	chr1:206798870-206842981	IL19	0.24
interleukin 34	chr16:70579895-70660682	IL34	0.40
interleukin 1 receptor type 1	chr2:102064544-102179874	IL1R1	0.64
interleukin 1 receptor type 2	chr2:101991844-102028544	IL1R2	4.88
interleukin 1 receptor accessory protein	chr3:190514051-190659750	IL1RAP	0.32
interleukin 1 receptor like 2	chr2:102186973-102240002	IL1RL2	0.84
interleukin 1 receptor antagonist	chr2:113107214-113134016	IL1RN	23.08
interleukin 4 receptor	chr16:27313668-27364778	IL4R	1.08
interleukin 6 receptor	chr1:154405193-154469450	IL6R	1.53
interleukin 17 receptor A	chr22:17084954-17115694	IL17RA	2.20
interleukin 17 receptor B	chr3:53846580-53865800	IL17RB	1.06
interleukin 17 receptor C	chr3:9917074-9933630	IL17RC	1.15
interleukin 17 receptor E	chr3:9902612-9916402	IL17RE	0.81
interleukin 18 binding protein	chr11:71998541-72005715	IL18BP	0.78
C-X-C motif chemokine ligand 1	chr4:73869393-73871242	CXCL1	6.93
C-X-C motif chemokine ligand 6	chr4:73836497-73849064	CXCL6	1.23
C-X-C motif chemokine ligand 16	chr17:4733526-4739922	CXCL16	1.12
C-C motif chemokine ligand 2	chr17:34255218-34257203	CCL2	0.64
C-C motif chemokine ligand 11	chr17:34285668-34288334	CCL11	0.68
interferon alpha and beta receptor subunit 1	chr21:33324477-33359862	IFNAR1	1.26
interferon alpha and beta receptor subunit 2	chr21:33229901-33265675	IFNAR2	0.39
interferon gamma receptor 2 (interferon gamma transducer 1)	chr21:33402896-33479348	IFNGR2	0.87
interferon regulatory factor 1	chr5:132481609-132490798	IRF1	0.82
interferon regulatory factor 3	chr19:49659569-49665875	IRF3	1.13
interferon regulatory factor 7	chr11:612553-615999	IRF7	0.35
interferon regulatory factor 9	chr14:24161053-24166565	IRF9	1.15
transforming growth factor beta 1	chr19:41301587-41353911	TGFβ1	1.34
transforming growth factor beta 2	chr1:218346235-218444619	TGFβ2	6.06
transforming growth factor beta 3	chr14:75958099-75982991	TGFβ3	0.78
transforming growth factor beta receptor 3	chr1:91680343-91906335	TGFβR3	0.55
nuclear factor kappa B subunit 1	chr4:102501329-102617302	NFKB1	0.90
nuclear factor kappa B subunit 2	chr10:102394110-102402529	NFKB2	2.31
NFKB inhibitor alpha	chr14:35401511-35404749	NFKBIA	1.72
NFKB inhibitor beta	chr19:38899700-38908893	NFKBIB	3.37
NFKB inhibitor delta	chr19:35887653-35902303	NFKBID	1.09
NFKB inhibitor epsilon	chr6:44258166-44265788	NFKBIE	1.47
NFKB inhibitor zeta	chr3:101827991-101861022	NFKBIZ	1.10
NFKB repressing factor	chrX:119588337-119605895	NKRF	1.89
NFKB inhibitor like 1	chr6:31546870-31558829	NFKBIL1	1.83
RELA proto-oncogene, NF-kB subunit	chr11:65653596-65663094	RELA	1.53
RELB proto-oncogene, NF-kB subunit	chr19:45001430-45038198	RELB	2.97
REL proto-oncogene, NF-kB subunit	chr2:60881521-60931610	REL	0.63
inhibitor of kappa light polypeptide gene enhancer in B-cells, kinase gamma	chrX:154541199-154565046	NEMO	1.20
conserved helix-loop-helix ubiquitous kinase	chr10:100188298-100229619	IKKα	1.45
inhibitor of kappa light polypeptide gene enhancer in B-cells, kinase beta	chr8:42271302-42332653	Ikk β	0.87
prostaglandin-endoperoxide synthase 2	chr1:186671791-186680427	PTGS2	1.32
epidermal growth factor receptor	chr7:55019021-55211628	EGFR	0.51

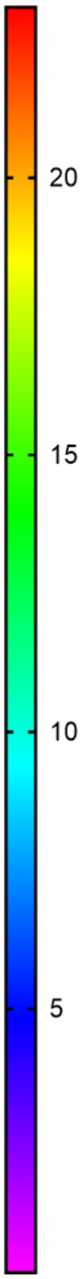

Figure S5. Transcriptional attenuation of inflammatory response to MG132 in classical HGPS fibroblasts. Heatmap of RNAseq data (ArrayExpress accession number: E-MTAB-5807) from HGPS fibroblasts treated with DMSO (vehicle control) or with 5 μM MG132 for 6 h. This analysis represents the fold change, in MG132-treated relative to DMSO-treated HGPS fibroblasts, of the most characteristic transcripts of the NF-κB pathway. ($n = 2$).

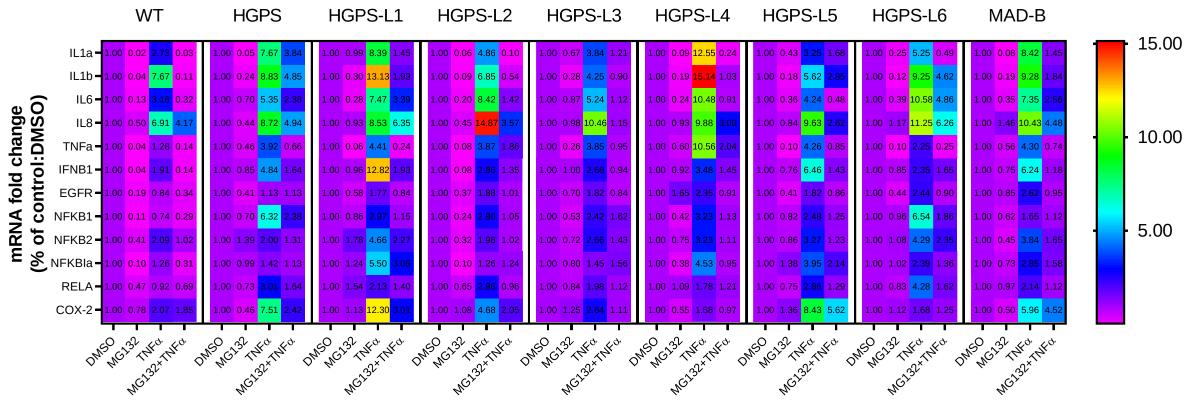

Figure S6. MG132 reduces the transcript levels of proinflammatory mediators and counteracts TNFα-induced inflammation. Quantitative real-time PCR using selected inflammatory genes expression arrays in culture supernatants of WT, HGPS, HGPS-like and MAD-B fibroblasts treated for 6 h with MG132 (500 nM), TNFα (10 ng/ml) alone and in combination or DMSO as a vehicle control.

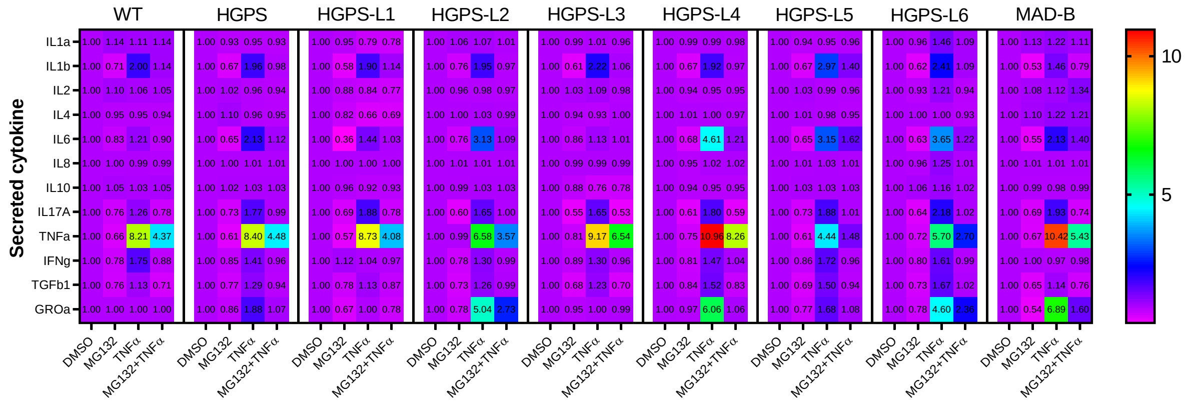

Figure S7. MG132 reduces the secretion of proinflammatory cytokines and alleviates TNFα-induced inflammation. Enzyme-Linked Immunosorbent Assay (ELISA) using multi-analyte ELISA arrays to measure inflammatory cytokines in culture supernatants of WT, HGPS, HGPS-like and MAD-B fibroblasts treated for 24 h with MG132 (500 nM), TNFα (10 ng/ml) alone and in combination or DMSO as a vehicle control.
